# Complete closed genomes of four *Burkholderia cepacia* complex clinical strains from cystic fibrosis patients

**DOI:** 10.1128/mra.00662-25

**Published:** 2025-08-21

**Authors:** Shari Tyson, Tamara Burdz, David Alexander, Paul Van Caeseele, Jennifer R. Tanner, Anne-Marie Bernier

**Affiliations:** 1 National Microbiology Laboratory, Public Health Agency of Canada, Winnipeg, Manitoba, Canada; 2 Cadham Provincial Laboratory, Winnipeg, Manitoba, Canada; 3 Department of Biology, Université de Saint-Boniface, Winnipeg, Manitoba, Canada; University of Maryland School of Medicine, Baltimore, Maryland, USA

**Keywords:** *Burkholderia cepacia* complex, closed genome, multiple chromosomes, plasmids

## Abstract

We report complete genome sequences of four *Burkholderia cepacia* complex isolates from cystic fibrosis patients which consist of three circular chromosomes and zero to two plasmids.

## ANNOUNCEMENT

The *Burkholderia cepacia* complex (BCC) includes species that can cause life-threatening disease in the lungs of cystic fibrosis (CF) patients (1), including *Burkholderia multivorans*, *Burkholderia contaminans*, and *Burkholderia cenocepacia* studied here as part of a larger project on chronic infections. Four pure cultures of clinical isolates collected between 2011 and 2018 from the throat or sputum of CF patients in Canada were referred to the Special Bacteriology Unit at the National Microbiology Laboratory in Winnipeg, Canada, for identification and multi-locus sequence typing (MLST). For DNA extractions, strains were cultured on Columbia blood agar plates at 35°C with 5% CO_2_ for 24 h, and species identified by MLST using seven housekeeping genes (https://pubmlst.org/organisms/burkholderia-cepacia-complex) (Table 1) (2, 3).

**TABLE 1:** Sequencing data and relevant features for the four BCC strains from NCBI Bioproject PRJNA852277

	NML110041	NML151013	NML140411	NML180963
Species	*B. cenocepacia*	*B. contaminans*	*B. multivorans*	*B. multivorans*
MLST^*a*^; CC^*b*^	ST 28; CC31	ST482; n/a	ST619; n/a	ST191; n/a
# bp total	7,971,239	8,666,185	6,308,387	6,455,318
# Replicons	4	5	3	4
Chr1	3,777,894	3,846,952	3,417,441	3,495,006
Chr2	3,216,338	3,158,005	2,341,839	2,259,977
Chr3	882,513	1,349,539	549,107	559,470
Pl 1	94,494	240,894	n/a^*c*^	140,865
Pl 2	n/a^*c*^	70,795	n/a^*c*^	n/a^*c*^
NCBI Ref Seq assembly	GCF_025643575.1	GCF_025643515.1	GCF_025643555.1	GCF_025643495.1
NCBI genbank accession	CP102475-CP102478	CP102462-CP102466	CP102472-CP102474	CP102458-CP102461
Genome coverage (hybrid assembly)	40	461	90	126
Sequencing depth (Illumina)	37	39	60	37
GC content (%)	66.5	66	67	67
# Illumina reads	1,051,336	1,013,244	1,284,952	1,458,206
# Illumina bases	314,760,500	335,507,561	381,722,916	431,094,110
# MinION reads	31,561	893,805	190,790	100,248
# MinION bases	157,806,889	3,695,880,523	788,950,699	1,017,283,192
MinION read length N50	9,597	6,182	7,800	19,410
SRA accessions MinION	SRR19887151	SRR19887148	SRR19887150	SRR19887147
SRA accessions Illumina	SRR19887156	SRR19887153	SRR19887155	SRR19887152
# Genes	7,390	7,978	5,732	6,000
# CDS (with protein)	7,151	7,750	5,572	5,824
# Complete rRNAs	18	18	15	15
# tRNA	73	71	66	67
Repeats (CRISPR)	0	0	0	0
Complete phage	3	1	3	4
ncRNA	5	4	4	4
Closest reference genome and NCBI ref seq accession	*B. cenocepacia* J2315 NC_011000.1– NC_011003.1	*B. contaminans* Strain ZCC NZ_CP042164.1–NZ_CP042168.1	*B. multivorans* ATCC BAA-247 NZ_CP009830.1–NZ_CP009832.1	*B. multivorans* ATCC 17616 NC_010084.1–NC_010087.1, NC_010070.1

					NCBI assembly accession	GCF_000009485.1	GCF_007724625.1	GCF_000959525.1	GCF_000018505.1

^*a*^ MLST: multi locus sequence type.

^*b*^ CC: clonal complex.

^*c*^ n/a : not applicable.

Unless noted, default parameters and manufacturer’s instructions were used for all procedures. DNA for Oxford Nanopore MinION (MK1B) sequencing was isolated using the MasterPure kit from Epicenter, quantified by Qubit, and size was verified on the Agilent TapeStation. Sequencing libraries (Oxford Nanopore Rapid Barcoding kit (SQK-RBK004)) were run on a FLO-MIN106 (v9.4 revD) flow cell on a MinION Mk1B instrument using MinION software (v19.06.8), and MinKNOW core (v.3.4.8). Guppy (v.3.2.2) fast algorithm was used for base calling, demultiplexing and filtering for reads of Q7+ quality, and length >1,000 bp (4). Long-read-only assemblies were generated using both Canu (v.1.7) and Unicycler (v.0.4.7) (5, 6).

Illumina short-read libraries were produced using DNA extracted with the QIAamp DNA Mini Kit (Qiagen, Cat No. 51306) and the Nextera XT library kit. Sequencing was performed using a 2 × 300v3 MiSeq kit. Fast QC (v.0.11.9) (https://www.bioinformatics.babraham.ac.uk/projects/fastqc/) confirmed high-quality reads (average Q30) and no further pre-processing was performed. Long reads and hybrid assemblies were compared in Mauve (v.20150226 build10) to confirm overall genome organization (7).

All assemblies were imported into GAP4 (Staden package v.4.11.2-r) where hybrid contigs were reviewed, and gaps closed using long read assemblies (either Canu or Unicycler) (8). Circularity was verified in GAP4 by comparing the sequence from the leading end of the contigs to the tail end. Where overlaps were found, BLAST searches confirmed circularity, and duplicate bases were removed. Circularized assemblies were checked for coverage by mapping short reads to them using Bowtie2 (v.2.3.4.1) and resulting .bam files were reviewed in GAP5 (Staden package v.1.2.14-r) for localized mis-assemblies, none of which were found.

Successive iterations of mapping with Bowtie2 and Pilon (v.1.20.1) polished the assemblies until no further changes were recognized (between 3 and 10 rounds) (9, 10).

The polished assembly of chromosome 1 for each strain was re-oriented to the origin (*dnaA* gene), and the remaining chromosomes were rotated to an alternate gene predicted by Prodigal using Circlator –fixstart (v.1.5.5) (11). Quast v.4.6.3 was used to generate assembly metrics relative to the closest presumed reference in NCBI (Table 1) (12). Busco (v.3.0.2) against the betaproteobacteria database was used as a measure of relative completeness of the assembly (13). Plasmids were found in three strains and identified as such by the lack of rRNA and tRNA genes. Phage was identified using Phaster (14).

Genomes were annotated with NCBI Prokaryotic Genome Annotation Pipeline (v.6.3) (15). Relevant metrics and data availability are presented in Table 1.

## Data Availability

This Whole Genome Shotgun project has been deposited at DDBJ/ENA/GenBank under the BioProject PRJN852277, and NCBI genbank accession numbers are in Table 1.
